# RiMINI – the influence of rifaximin on minimal hepatic encephalopathy (MHE) and on the intestinal microbiome in patients with liver cirrhosis: study protocol for a randomized controlled trial

**DOI:** 10.1186/s13063-016-1205-8

**Published:** 2016-02-29

**Authors:** Christian Schulz, Kerstin Schütte, Siegfried Kropf, Friedhelm C. Schmitt, Riccardo Vasapolli, Leon M. Kliegis, Antonia Riegger, Peter Malfertheiner

**Affiliations:** Department of Gastroenterology, Hepatology and Infectious Diseases, Otto-von-Guericke University Magdeburg, Leipziger Str. 44, 39120 Magdeburg, Germany; Institute for Biometry and Medical Informatics, Otto-von-Guericke University, Magdeburg, Germany; Department of Neurology, Otto-von-Guericke University, Magdeburg, Germany; Helmholtz Centre for Infection Research, Microbial Interactions and Processes (MINP) Research Group, Braunschweig, Germany

**Keywords:** Hepatic encephalopathy, Minimal hepatic encephalopathy, Liver cirrhosis, Rifaximin

## Abstract

**Background:**

Hepatic encephalopathy (HE) is a clinically significant complication of liver cirrhosis impacting on the patients’ quality of life. Minimal hepatic encephalopathy (MHE) is diagnosed by psychometric tests, found in up to 80 % of patients with liver cirrhosis and carries a high risk of progression to overt HE. Continuous therapy with rifaximin in combination with lactulose significantly reduces the risk of overt HE, recurrence of HE and HE-related hospitalizations in randomized, double-blind, placebo-controlled clinical trials. Rifaximin is approved for the therapy of overt HE in Germany. Treatment with lactulose has been shown to improve cognitive functions in patients with liver cirrhosis. Data from prospective clinical trials comparing the efficacy of rifaximin alone against a combination of rifaximin and lactulose in the treatment of MHE are scarce. Changes in the microbiome of the upper and lower gastrointestinal tract as a result of therapy with rifaximin have not yet been addressed in clinical studies.

**Methods and design:**

RiMINI is a monocentric exploratory pilot study on 60 patients with MHE as assessed by critical flicker frequency (CFF). Additionally, visual evoked potentials’ (VEP) testing, electroencephalography (EEG) and psychometric testing (NCT-A) will be carried out. Patients will be randomized to treatment either with rifaximin alone (550 mg twice daily (bid) continuously for a period of 3 months) or with rifaximin (550 mg bid continuously) in combination with lactulose (30–60 ml daily) for 3 months. An esophagogastroduodenoscopy (EGD) will be performed at baseline, at the end of treatment and 6 and 12 weeks after the end of treatment to obtain gastric and duodenal biopsies and aspirates. The samples will be analyzed for their content of specific bacterial taxae by applying next generation sequencing (NGS) after rRNA isolation to identify the microbiome of the stomach and duodenum, and of the gut, in patients with liver cirrhosis and MHE before and after therapy.

**Discussion:**

Differences of the effect of antibiotic therapy with rifaximin alone or in combination with lactulose on the clinical course of MHE are assessed.

**Trial registration:**

The trial was registered as DRKS00006359  on March 17th 2015, with the universal trial number U1111-1163-9410 and with EudraCT2013-004414-18.

## Background

Hepatic encephalopathy (HE) is defined as an unspecific brain function disorder, due to either failure of (cirrhotic) liver function or to port osystemic shunting and thus by-passing the functional parenchyma. For an accurate diagnosis of HE, other brain diseases have to be properly excluded. HE is characterized by cognitive, psychiatric and/or motoric impairment [1] leading to changes in the circadian rhythm, atmospheric fluctuations, phased loss of concentration, increased forgetfulness, irritability, and somnolence up to hepatic coma [2]. Subclinical neurological deficits are defined as minimal hepatic encephalopathy (MHE). The presence and degree of (M)HE can only measured by psychometric tests and clinically apparent deficits, graded by the West Haven criteria [1, 3]. Clinically apparent (i.e., overt) HE is estimated to be present in 30–45 % of patients with liver cirrhosis and leads to frequent hospitalization, impairment of health-related quality of life (HRQL) and ability to work [4]. Up to 80 % of cirrhotic patients present with MHE with cognitive dysfunction in special diagnostic testing [5, 6, 7]. In patients with MHE activities of daily life are affected resulting, among other problems, in an increased risk for accidents when driving and navigating [8]. The clinical manifestations of HE in each stage are potentially reversible by adequate therapeutic management. MHE is currently under-diagnosed in patients with liver cirrhosis and carries a high potential for progression to overt HE. Current therapies are directed at the reduction of ammonia production from the gastrointestinal tract by administration of the non-absorbable disaccharide lactulose and the non-absorbable antibiotic rifaximin [9–18].

### Rationale for the explorative study

Continuous therapy with rifaximin in combination with lactulose significantly reduces the risk of overt HE, recurrence and HE-related hospitalization in randomized double-blind placebo-controlled clinical trials [1–3]. Rifaximin is approved for the therapy of *overt* HE in Germany. Therapy with lactulose has been shown to improve cognitive functions in patients with liver cirrhosis. So far, whether monotherapy with rifaximin is as effective as combination therapy with rifaximin and lactulose in the treatment of MHE, has not been addressed in a prospective clinical trial.

Rifaximin is a minimally absorbed gut-selective antibiotic and thus impacts on the intestinal microbiome. The role of the intestinal microbiome in the pathophysiology of HE is still little understood. To our knowledge, changes in the microbiome of the upper gastrointestinal (GI) tract as result of therapy with rifaximin have not yet been addressed in clinical studies and studies addressing the persistence of changes of the intestinal microbiome are scarce [19].

## Methods and design

RiMINI is a monocentric exploratory pilot study on 60 patients with MHE as assessed by critical flicker frequency (CFF) analysis. Additionally, visual evoked potentials’ (VEP) testing, electroencephalography (EEG) and a psychometric test (NCT-A) will be carried out. The patients will be randomized to treatment either with rifaximin alone 550 mg twice daily (bid) continuously for a period of 3 months or to continuous treatment with 550 mg rifaximin bid in combination with lactulose 30–60 ml daily (to pass 2–3 semisoft stools/day) for 3 months. An esophagogastroduodenoscopy (EGD) will be performed at baseline, at the end of antibiotic treatment, as well as 6 and 12 weeks after the end of treatment to obtain gastric and duodenal biopsies and aspirates will be taken. The samples will be analyzed for their specific bacterial taxae by applying next generation sequencing (NGS) after ribonucleic acid (RNA) isolation. Additionally, stool samples will be taken at the same time points. Prevalence of *Helicobacter pylori*, which is the predominant gastric microbe, will be assessed by histology, rapid urease test and serology at study entry. For clinical assessment, quantification of MHE and therapeutic effects, CFF, EEG, VEP and psychometric tests are assessed weekly during the first month and every 4 weeks from the beginning of the second month until the end of the post-treatment follow-up.

**Fig. 1:**
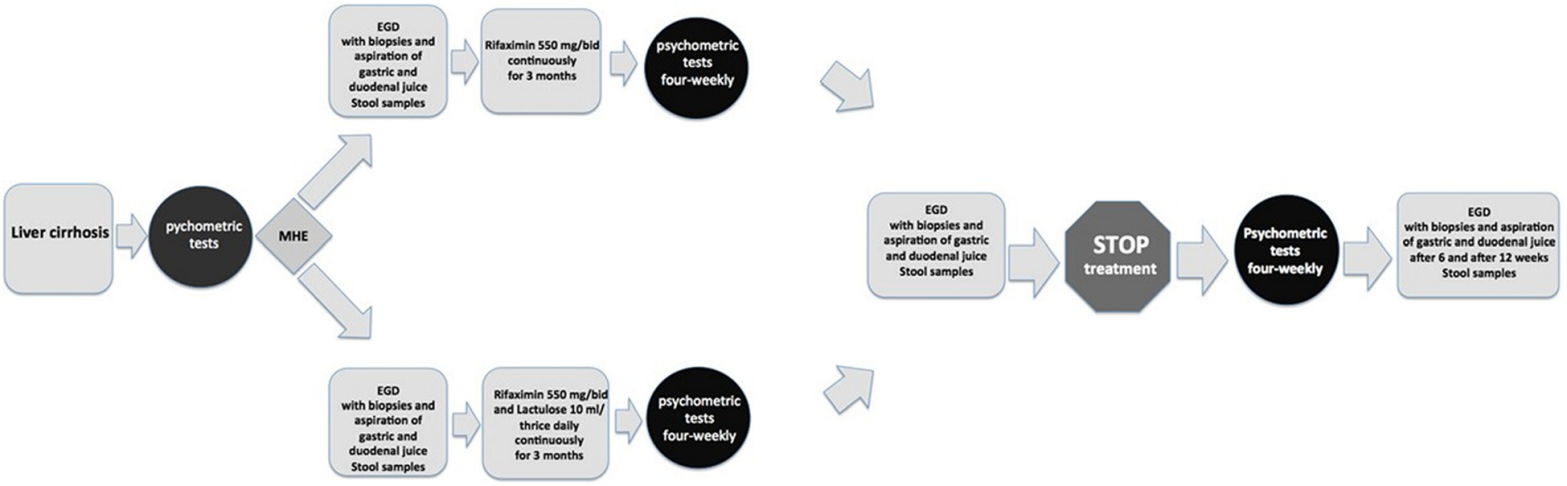
Study flow chart

Changes in the gut microbiome from antibiotic therapy with rifaximin alone or rifaximin with lactulose are analyzed comparing pre- and post-antibiotic time points. The follow-up data will show whether the change in the gut microbiome is persistent or transient. We will compare the effect of antibiotic therapy with rifaximin alone with a combination therapy with lactulose on the clinical course of MHE. As planned, NGS methods (Illumina sequencing, Illumina, San Diego, CA, USA) will be used to characterize the composition and changes of both stomach and small bowel microbiomes. Bacterial ribosomal ribonucleic acid (rRNA) will be isolated from aspirates, biopsies and feces and then stored in RNAlater at −80 °C. The samples will be analyzed by NGS methods as described below with the aim of identifying the microbiome of the stomach and the small bowel, as well as of the large bowel, in patients with liver cirrhosis and MHE and its changes before and after therapy (Fig. 1).

### Methods for studying intestinal microbiota

Samples will be taken following a strict algorithm. Aspirates will be collected using a sterile, straight endoscopic retrograde cholangiography (ERC)-catheter via a standard gastroscope (Olympus 180). After collecting the aspirate from the duodenum (pars descendens), biopsies will be taken from this area after removal of the catheter. Afterwards gastric juice will be aspirated using a second sterile catheter. Biopsies will be taken from the antral region of the stomach for RNA extraction. Additional biopsies for histopathological examination, rapid urease testing and culturing of *H. pylori*, if infected, will be taken from the duodenum, the gastric antrum, the angulus and the gastric corpus. After completion of the endoscopic procedure, a saliva sample will be taken directly from the oral cavity of the patients. All samples determined for RNA extraction will be stored directly in 1 ml RNAlater (Sigma-Aldrich, St. Louis, MO, USA) and kept at room temperature for at least 4 hours (maximum 8 hours). Afterwards, samples will be stored at −80 °C. RNA will be extracted from the aspirates, feces and biopsies using the RNeasy Mini Kit following the manufacturer’s instructions (Qiagen, Hilden, Germany). In summary: the biopsies will be relocated from the RNAlater into buffer solution as specified, whereas the liquids will be centrifuged for 10 minutes at 15,000 rpm and 4 °C and resuspended into buffer solution. The solutions will be replaced in Lysing Matrix E tubes and the cells will be lysed and homogenized using a Fast Prep™-24 instrument (MP Biomedicas, Solon, OH, USA) for 2 × 45 seconds, interrupted by a 1-minute ice cooling break. Additionally, we will complement a second digestion of deoxyribonucleic acid (DNA) on the columns using the RNase-Free DNase Set (Quiagen, Hilden, Germany) working with 70 μl RDD buffer and 10 μl DNase for each sample for 8 minutes. Finally, the RNA will be eluted in 32 μl RNase-free water.

To synthesize first-strand complementary deoxyribonucleic acid (cDNA) the SuperScript® III First-Strand Synthesis System (Invitrogen™, Carlsbad, CA, USA) will be used following the manufacturer’s instructions.

The V1–V2 region of the 16S rRNA gene will be amplified using primers based on 27F and 338R primers. The primers include sequences complementary to the Illumina-specific adaptors to the 5′ends that are used in the course. Amplification will be performed in a total volume of 50 μl with 5x PrimeSTAR TM buffer (Clontech Laboratories, Mountain View, CA, USA). Each deoxyncleoside triphosphate will be contained at a concentration of 2.5 mM and each primer will be contained at a concentration of 0.2 μM. One microliter of cDNA template will be used with 0.5 μl PrimeSTAR TM HS DNA polymerase (2.5 U, Clontech Laboratories, Mountain View, CA, USA). The polymerase chain reaction (PCR) will be started with an initial denaturation step of 95 °C for 3 minutes, followed by 15 cycles of denaturation at 98 °C for 10 seconds, annealing at 55 °C for 10 seconds and extension at 72 °C for 45 seconds. Non-template control (water) and positive control (*Staphylococcus aureus* DSM3463) were used to exclude contaminants and systematic errors. A second PCR will be performed using 1 μl reaction product under the same conditions for 20 cycles using PCR primers designed to integrate the sequence of the specific Illumina multiplexing sequencing primers and index primers. Negative control (water) will document the absence of contaminants.

Following the third PCR amplicons will be verified by agarose gel electrophoresis and purified using Macherey-Nagel 96-well plate purification kits (Macherey-Nagel, Düren, Germany) following the manufacturer’s instructions. The products will be quantified with the Quant-iT PicoGreen double-stranded deoxyribonucleic acid dsDNA) reagent and the associated kit (Invitrogen, Darmstadt, Germany).

Amplicon libraries will be prepared by pooling equimolar ratios of amplicons (200 ng/sample). After combining five libraries with unique barcode primers and different index primers, a second precipitation step will be performed to remove any contaminants. The supernatant will be removed, air dried and resuspended in 30 μl of double-distilled water. The product will be separated on a 2 % agarose gel. PCR products of the correct size will be extracted and recovered using the QIAquick gel extraction kit (Qiagen, Hilden, Germany). Libraries will be sent for 150-nt paired-end sequencing on a GAIIX Genome Analyzer (Illumina, San Diego, CA, USA).

The sequencing analyses will be followed by bioformatical analyses using Primer7 (Primer-E Ltd, Lutton, United Kingdom).

### Primary objective

This study primarily aims at the analysis of the efficacy of MHE therapy with rifaximin alone or in combination with lactulose on the improvement of neuropsychometric and neurophysiological changes.

### Secondary objective

Composition and changes in the composition of the intestinal microbiome of the upper GI tract, as well as of the large bowel, before, during and after therapy of MHE with either a combination of lactulose and rifaximin or rifaximin monotherapy are the focus of further analyses.

### Eligibility/Description

Patients of either sex are eligible for this trial provided they meet all inclusion and exclusion criteria.

### Inclusion criteria

Individuals who are suitable to participate as determined by the outcome of medical history, physical assessment, and clinical judgment of the investigator can be included into the study.

In order to participate in this study, all subjects must meet all the following inclusion criteria in the “All subjects” section below.

### All subjects

Men and women in the age range 18–90 years at the time of enrollment and who are mentally competent, willing and able to understand the nature and risks of the proposed study, and able to sign the consent form prior to study entry. The individuals must have a projected life expectancy of 6 months or longer and must be able to comply with all study procedures and requirements. Women with childbearing potential must have a negative pregnancy test during the screening period. The diagnosis of liver cirrhosis is needed, established either by histology or by typical signs on transabdominal ultrasound in combination with signs of portal hypertension (ascites, enlarged spleen, fundic or esophageal varices), and the presence of MHE has to be confirmed. To confirm the diagnosis of MHE the following tests will be performed: number-connection test A (NCT-A), critical flicker frequency analysis (CFF), electroencephalography (EEG), and visual evoked potentials (VEP) whereby the final confirmation of MHE depends on the CFF result (CFF <39 Hz). The additional neurophysiological and neuropsychometric tests will be analyzed concerning diagnostic utility alone or in combination.

### Exclusion criteria

Individuals with any of the following medical conditions are excluded from participation in the trial:

Any documented underlying allergic condition against rifaximin or lactulose, underlying blindness *or* eye axis deviation *or* red-green color blindness. The presence of overt HE rules out participation in the study. Antibiotic treatment within 28 days before study entry or during the study constitutes an exclusion criterion. Individuals who are not sufficiently healthy as determined by medical history, physical assessment, and clinical judgment of the investigator are excluded. Individuals who are unable to follow the required study procedures for the whole period of the study and/or who have behavioral or cognitive impairment or psychiatric disease that, in the opinion of the investigator, may interfere with the subject’s ability to participate in the trial, are excluded. Individuals who are expected to be hospitalized (except for endoscopic therapy of esophageal varices) or undergo surgery during the study period or who have participated in another clinical study within 30 days prior to enrollment into the study are not allowed to participate. Individuals with ongoing drug or alcohol abuse that, in the opinion of the investigator, would interfere with the subjects’ safety or the evaluation of the study objectives, and individuals who are member of the research staff or have relatives who are member of the research staff, are excluded. Women with childbearing potential who have a positive pregnancy test (positive urine or serological (beta HCG)), and pregnant or lactating women are not allowed to participate as are women who are unwilling to use an acceptable method of birth control up to visit 4. Acceptable methods of birth control include (1) established use of oral, injected or implanted hormonal methods of contraception: placement of an intrauterine device or intrauterine system, (2) barrier methods of contraception: condom or occlusive cap (diaphragm or cervical/vault caps) with spermicidal foam/gel/film/cream/suppository, (3) male sterilization (with the appropriate post-vasectomy documentation of the absence of sperm in the ejaculate), (4) true abstinence from heterosexual sexual intercourse: when this is in line with the preferred and usual lifestyle of the subject. (Periodic abstinence (e.g., calendar, ovulation, symptothermal, post-ovulation methods) and withdrawal are not acceptable methods of contraception). Individuals with a medical history of any illness that may, in the opinion of the investigator, pose additional risk to the subject due to participation in the study, are also excluded.

### Prior and concomitant medication

Patients may receive concomitant therapy during the study as required. Any concomitant medication at baseline as well as any changes made in concomitant medication within the study period (until the last follow-up examination) will be recorded in the electronic Case Report Form (eCRF). All concomitant therapy will be recorded using generic names. The necessity to use antibiotic therapy besides rifaximin leads to exclusion from this study.

### Schedule of evaluations/Estimated timelines

This study consists of three phases: (1) the screening phase, (2) the treatment phase and (3) the follow-up phase (Table 1).

**Table 1:** Clinical treatment plan

	Inclusion into study	Screening	Assignment to treatment group	Treatment phase	EOT	6 weeks after EOT	12 weeks after EOT = End of study
Time		Day −15 –day 0	0 (visit 0)	Days 1–90 (visits 1–3) −3d to +3d	Visit 3	Day 129– day 135 (visit 5) −3d to +3d	Day 171– day 177 (visit 6) −3d to +3d
Informed consent	x						
Demographics		X					
Medical history concomitant medication		X		x	X	x	X
Physical exam, vital signs, weight		X	x	x	X	x	X
ECG		X					
ECOG performance status and QOL assessment		X	x	x	X	x	X
Blood tests including pregnancy test		X	x	x	X	x	X
EGD with biopsies			x		X	x	X
	Inclusion into study	Screening	Assignment to treatment group	Treatment phase	EOT	6 weeks after EOT	12 weeks after EOT = End of study
Time		Day −15–day 0	0 (visit 0)	Days 1–90 (visits 1–3) −3d to +3d	Visit 3	Day 129– day 135 (visit 5) −3d to +3d	Day 171– day 177 (visit 6) −3d to +3d
*H. pylori* serology			x				
Stool samples			x	x	x	x	x
Rapid urease test			x				
Assessment of neurocognitive function (NCT-A, CFF, VEP, EEG)		X		Days 1–28 weekly, then every 4 weeks	>x	x	x

*CFF* critical flicker frequency, *ECG* electrocardiogram *ECOG* Eastern Cooperative Oncology group, *EEG* electroencephalogram, *EGD* esophagogastroduodenoscopy, *EOT* end of treatment, *NCT-A* number-connection test A, *QOL* quality of life, *VEP* visual evoked potentials

### Recruitment

Patients will be recruited in the hospital department where they are treated for their primary disease. This will usually be the Department of Gastroenterology, Hepatology and Infectious Diseases of the University of Magdeburg, Germany. Patients will be informed by the investigator or a sub-investigator about the scope and the goals of this trial on the basis of the study-specific patient information leaflet and are then invited to participate. Any study-related measure (including diagnostic screening procedures) will only take place after the patient has personally signed and dated the study-specific informed consent form. Patients will have at least 24 hours to consider their participation in this trial before signing the informed consent form.

### Randomization procedure

The randomization will be performed with the clinical database software secuTrial that is also used for the data management in the study. The two arms will be randomized in a 1:1 ratio using the minimization method described by Pocock and Simon with a stratification for age and gender.

### Blinding

Due to the study concept (pills versus pills + liquids) a (double-)blinded concept appeared inoperable: therefore, the study will be performed as an open label study. The use of objective neuropsychometric measurements without learning curves ensures a maximum decrease of confounding effects.

### Withdrawal and replacement criteria for treatment

Every patient has the right to refuse further participation in the study at any time and without providing any reasons. A patient’s participation will be terminated immediately upon their request. The investigator should seek to obtain the reason and record this in the eCRF. This study is designed as an intention-to-treat (ITT) study.

### Safety assessment

Patient safety will be assessed at every visit until the end of treatment. They will be monitored for adverse events (AEs) using NCI-CTCAE V4.0 criteria. All AEs are to be documented in the eCRF. In cases of serious adverse events (SAEs), the event is also to be documented on the SAE form provided by the sponsor. The SAE management will be conducted by the Clinical Study Center. The processes are described in a separate SAE Manual. Annually, a development update safety report (DSUR) must be submitted to the competent authority (BfArM).

### Statistical analysis

All demographic and baseline variables are presented in descriptive statistics stratified by treatment arm in the full analysis set (FAS, all randomized patients). Exploratory tests, according to the level of the respective variables, will search for differences between both arms which might occur despite randomization. The primary target variable is compared between both treatment arms. The odds ratio for treatment success is calculated between both treatment arms together with the corresponding two-sided 95 % confidence interval. If the confidence interval is completely above or below the threshold of 1, the superiority of the corresponding treatment arm with larger success rates can be concluded. If the confidence interval covers the threshold of 1, then the lower and upper confidence limits give limits which can be used in non-inferiority statements. This analysis is performed primarily in the modified full analysis set (MFAS, all randomized patients but excluding those with missing CFF tests) according to the ITT principle. Missing values are imputed by the last observation carried forward principle.

Secondary analyses for the primary target variable include (1) logistic regression analysis with treatment arm, sex, age and baseline value of CFF as co-variables, (2) analog analyses in the per protocol set (PPS, patients of the MFAS excluding those with severe protocol violations), (3) analog analyses for the result of CFF at end of follow-up, (4) description of the results of the psychometric tests (mean and standard deviation) per treatment arm and comparison in two-sample *t* tests (both at end of treatment and end of follow-up).

The secondary efficacy variables are compared between the treatment arms in analysis of covariance (ANCOVA) models with treatment arm, sex, age and respective baseline variable as factors or co-variables, respectively.

Safety analysis is done in the safety set (SS, patients receiving at least one treatment) in descriptive and exploratory analyses. Laboratory variables and rates of SAEs are compared between both treatment arms using the Mann-Whitney *U* test and Fisher’s exact test.

### Power analysis

This is an exploratory pilot study as so far data on the monotherapeutic effect of rifaximin in the treatment of MHE are scarce. The results will give the basis for planning a later confirmatory multicenter trial. With the given sample sizes the proof of differences between the two treatment options, or of acceptable non-inferiority margins, can be expected only if the additional of lactulose drastically improves the success rates of the rifaximin treatment.

### Ethics

The planning and conduct of this clinical study are subject to national laws. The study will be conducted in accordance with the protocol and the ethical principles that have their origin in the Declaration of Helsinki and the International Conference on Harmonization guidelines for Good Clinical Practice (ICH-GCP) [20, 21].

The study protocol and any amendments have been reviewed and approved by the ethic committee of the Otto-von-Guericke University of Magdeburg, Germany, and by the competent authority, BfArM, before implementation.

### Data handling, data processing, monitoring and auditing

Data required according to this protocol are to be recorded in the eCRFs in a timely manner. A Data Manual (DM) will be maintained specifying all relevant aspects of data processing for the study (including data validation, cleaning, correcting, releasing).

For data coding (e.g., AEs, baseline findings, medication, medical/surgical history), internationally recognized and accepted dictionaries will be used.

All monitoring activities will be overseen by the coordinating study bureau. A member of the sponsor’s (or designated chief research officer’s (CRO’s) quality assurance unit may visit the investigator annually in order to audit the performance of the study at the study site and the study documents originating there.

In addition, inspections by health authority representatives – including foreign authorities – and Institutional Ethical Committees (IECs) and Institutional Review Boards (IRBs) are possible at any time.

## Discussion

Rifaximin treatment for patients with overt HE has been shown to be effective in the amelioration of cognitive function and in the prevention of recurrence of overt HE [14]. In patients with MHE rifaximin significantly improves cognitive function and HRQL [13, 14]. However, more than 90 % of patients with HE treated in prospective studies received a combination therapy of rifaximin and lactulose. Only a single prospective trial with a limited number of subjects addressed the effect of rifaximin on MHE [13]. The primary objective of our study, therefore, is to analyze the efficacy of rifaximin alone or in combination with lactulose on the improvement of neuropsychometric and neurophysiological changes in patients with MHE. Although current therapeutic concepts in the treatment of HE are directed to reduce bacterial ammonia production by antibiotic treatment, the role and contribution of the upper-GI microbiota in the pathogenesis of HE remain poorly understood. The secondary objective of this study aims at evaluating the microbial diversity and the composition of the microbiota in the upper GI tract, and in the stool, in cirrhotic patients with and without MHE before, during, and after therapy to assess the degree of changes and whether they are transient or persistent.

## Trial status

This trial is currently recruiting patients. The first patient was randomized on 24 April 2015.

## Abbreviations

AE: adverse event
cDNA: complementary deoxyribonucleic acid
CFF: critical flicker frequency
CRO: chief research officer
DM: Data Manual
DNA: deoxyribonucleic acid
dsDNA: double-stranded deoxyribonucleic acid
DSUR: development update safety report
eCRF: electronic Case Report Form
EEG: electroencephalography
EGD: esophagogastroduodenoscopy
ERC: endoscopic retrograde cholangiography
FAS: full analysis set
HE: hepatic encephalopathy
HRQL: health-related quality of life
IEC: Institutional Ethical Committee
IRB: Institutional Review Board
MFAS: modified full analysis set
MHE: minimal hepatic encephalopathy
NCT-A: number- connection test A
NGS: next generation sequencing
PCR: polymerase chain reaction
PPS: per protocol set
RNA: ribonucleic acid
rRNA: ribosomal ribonucleic acid
SAE: severe adverse event
SS: safety set
VEP: visual evoked potentials
